# Influence of metabolic syndrome factors and insulin resistance on the efficacy of ezetimibe/simvastatin and atorvastatin in patients with metabolic syndrome and atherosclerotic coronary heart disease risk

**DOI:** 10.1186/s12944-015-0075-5

**Published:** 2015-09-04

**Authors:** Jeffrey B. Rosen, Christie M. Ballantyne, Willa A. Hsueh, Jianxin Lin, Arvind K. Shah, Robert S. Lowe, Andrew M. Tershakovec

**Affiliations:** Clinical Research of South Florida, Coral Gables, FL USA; Baylor College of Medicine and Methodist DeBakey Heart and Vascular Center, Houston, TX USA; Wexner Medical Center, The Ohio State University, Columbus, Ohio, USA; Merck & Co., Inc., Kenilworth, NJ USA

## Abstract

**Background:**

Metabolic syndrome (MetS) and insulin resistance (IR) are increasing in prevalence, are associated with higher risk for coronary heart disease (CHD), and may potentially influence the responses to lipid-altering drug therapy. This study evaluated the effects of MetS factors (abdominal obesity, depleted high-density lipoprotein cholesterol [HDL-C], and elevated triglycerides, blood pressure, and fasting glucose) and IR on ezetimibe/simvastatin and atorvastatin treatment efficacy in patients with MetS.

**Methods:**

This post-hoc analysis of a multicenter, 6-week, double-blind, randomized, parallel group study of 1128 subjects with hypercholesterolemia, MetS, and moderately high/high CHD risk evaluated the effects of baseline MetS factors/IR on percent change from baseline in lipids, apolipoproteins, and high-sensitivity C-reactive protein (hs-CRP), after treatment with the usual starting doses of ezetimibe/simvastatin (10/20 mg) versus atorvastatin (10 mg, 20 mg) and next higher doses (10/40 mg versus 40 mg).

**Results:**

Ezetimibe/simvastatin and atorvastatin efficacy was generally consistent across MetS factor/IR subgroups. Ezetimibe/simvastatin produced greater incremental percent reductions in LDL-C, non-HDL-C, apolipoprotein B, total cholesterol, and lipoprotein ratios for all subgroups, and larger percent increases in HDL-C and apolipoprotein AI for all but non-obese and HDL-C ≥40 mg/dL subgroups than atorvastatin at the doses compared. Triglycerides, very-LDL-C, and hs-CRP results were more variable but similar between treatment groups.

**Conclusion:**

The magnitude of lipid-altering effects produced by each treatment regimen was generally similar across all MetS and IR subgroups. Ezetimibe/simvastatin produced greater percent reductions in most lipid fractions than atorvastatin at the dose comparisons studied, and all treatments were generally well tolerated. (Registered at clinicaltrials.gov: NCT00409773)

## Background

Metabolic syndrome is a cluster of factors that substantially increase the risk for atherosclerotic cardiovascular disease and diabetes [1, 2]. The estimated prevalence of metabolic syndrome found in most industrialized countries is 20-30 % [3–5]. For the United States, the National Health and Nutrition Examination Survey (NHANES) age adjusted data from 2003 to 2006 estimated that 34 % of Americans had metabolic syndrome, representing a 10 % increase over the previous 10–15 years [6]. The increased prevalence of metabolic syndrome is a worldwide public health issue, driven primarily by higher rates of obesity and an aging population [3, 4]. These observations highlight the urgent need for effective strategies to treat the underlying causes of metabolic syndrome, including weight loss, increased physical activity, and management of factors responsible for elevated cardiometabolic risk.

Recently defined criteria used to diagnose metabolic syndrome include abdominal obesity (waist circumference), dyslipidemia (reduced levels of high-density lipoprotein cholesterol [HDL-C] and high triglycerides), elevated blood pressure, and elevated fasting glucose [7]. Several national and international organizations have provided guidance for the management of metabolic syndrome and associated cardiovascular risk and for those patients with dyslipidemia [1, 8–12] Many international guidelines recommend specific treatment targets for low-density lipoprotein cholesterol (LDL-C), non-HDL-C, and apolipoprotein (apo) B levels based on cardiovascular risk; however the 2013 American College of Cardiology (ACC)/American Heart Association (AHA) guidelines focus on the identification of patient groups most likely to benefit from treatment with high-intensity statins that will result in LDL-C lowering by ≥50 % or by 30 to <50 %, respectively [12]. Obesity and insulin resistance modulate the normal pattern of lipid metabolism which promotes the development of atherogenic dyslipidemia, including higher levels of triglyceride-rich very-low density lipoprotein (VLDL), greater numbers of apo B-containing small-dense LDL particles, and increased clearance of circulating HDL [13]. These metabolic changes may potentially influence the efficacy of lipid-altering drugs, and it is therefore important to determine the effects of metabolic syndrome factors and insulin resistance on treatment.

The Vytorin in Metabolic Syndrome (VYMET) study was conducted in patients with metabolic syndrome, hypercholesterolemia, and either moderately high or high coronary heart disease (CHD) risk. Primary analysis of this study showed that after 6 weeks of treatment, ezetimibe/simvastatin produced significantly greater improvements in LDL-C and other key lipid parameters than atorvastatin at the doses compared [14]. The objective of this current *post hoc* analysis was to evaluate the efficacy of treatment in the presence or absence of specific metabolic syndrome factors and insulin resistance tertiles.

## Methods

### Trial design

This *post hoc* analysis evaluated data from the previously reported multicenter, randomized, double-blind, 5-arm, parallel-group VYMET study (NCT00409773; protocol number 107) that was designed to assess the lipid-altering efficacy of ezetimibe/simvastatin and atorvastatin in subjects with metabolic syndrome and elevated cardiovascular risk [14]. The protocol was approved by the institutional review board or ethics committee of each participating study site, and all patients provided written informed consent.

### Subjects and therapy

Details of the study have been described previously [14]. In short, participants eligible for inclusion in this study were men and women from 18 to 79 years old with a diagnosis of metabolic syndrome, hypercholesterolemia, and at moderately high or high risk of CHD. Metabolic syndrome was defined according to the 2005 AHA/National Heart Lung and Blood Institute criteria as having three or more of the following five characteristics; abdominal obesity (waist circumference of ≥102 cm [≥40 in] for men or ≥88 cm [≥35 in] for women); triglycerides ≥150 mg/dl; HDL-C <40 mg/dl in men or <50 mg/dl in women; blood pressure ≥130 mm Hg systolic blood pressure or ≥85 mm Hg diastolic blood pressure or on antihypertensive drug treatment in a subject with a history of hypertension; and fasting glucose ≥100 mg/dl or on drug treatment for elevated glucose [1]. High-risk subjects with CHD or other forms of atherosclerotic vascular disease (AVD, including peripheral arterial disease, atherosclerotic aortic disease, and carotid artery disease [15]) were required to have baseline LDL-C levels ≥70 mg/dl. High-risk subjects without AVD (diabetes or multiple risk factors and a 10-year risk level for CHD >20 %) and moderately high-risk subjects (10-year risk level for CHD of 10 to 20 %) were required to have baseline LDL-C levels ≥100 mg/dl. Eligible subjects who had not received treatment with lipid-lowering agents including over-the-counter dietary supplements of fish oils containing >1000 mg/day of EPA + DHA, rice yeast extract, Cholestin, bile-acid sequestrants, HMG-CoA reductase inhibitors, ezetimibe, ezetimibe/simvastatin; and niacin (>200 mg/day) within 6 weeks (8 weeks for fibrates) prior to the first study visit and were at high risk for CHD, or those receiving lipid-lowering therapy who were at moderately high risk for CHD, were randomized to treatment with the recommended starting doses of ezetimibe/simvastatin (10/20 mg) versus atorvastatin (10 and 20 mg) or next higher doses of ezetimibe/simvastatin (10/40 mg) versus atorvastatin (40 mg) for 6 weeks.

### Statistical analysis

Consistent with the main study, this *post hoc* subgroup analysis included all randomized subjects with a baseline and at least one post-baseline evaluation. Subgroups included the presence or absence of individual metabolic syndrome factors; abdominal obesity (yes, no), triglycerides ≥ 150 mg/dl (yes, no), HDL-C < 40 mg/dl for males or <50 mg/dl for females (yes, no), systolic blood pressure ≥130 mmHg or diastolic blood pressure ≥85 mmHg (yes, no), fasting glucose ≥100 mg/dl (yes, no); and insulin resistance tertiles (estimated using the Homeostasis Model Assessment of Insulin Resistance (HOMA-IR) score [16]: fasting serum insulin (mU/l) X fasting glucose (mmol/l)/22.5 [<2.72, 2.72-4.81, >4.81]). Treatment efficacy of ezetimibe/simvastatin 10/20 mg versus atorvastatin 10 mg and 20 mg, and ezetimibe/simvastatin 10/40 mg compared with atorvastatin 40 mg was evaluated within each subgroup using an analysis of variance (ANOVA) model with terms for subgroup, baseline risk stratum, treatment, and treatment by subgroup interaction. Efficacy endpoints included percent change from baseline in LDL-C, HDL-C, non-HDL-C, total cholesterol, VLDL-C, triglycerides, apo B, apo AI, lipid/lipoprotein ratios (total cholesterol:HDL-C, LDL-C:HDL-C, apo B:apo AI, non-HDL-C:HDL-C) and high-sensitivity C-reactive protein (hs-CRP) after 6 weeks of therapy. Least squares mean was determined for LDL-C, HDL-C, non-HDL-C, total cholesterol, VLDL-C, apo B, apo AI, and lipid/lipoprotein ratios, and estimates of between-treatment differences (ezetimibe/simvastatin minus atorvastatin) and corresponding 95 % confidence intervals (CIs) were determined using the ANOVA model. Median values were determined for triglycerides and hs-CRP, and estimates of between-treatment group differences and corresponding 95 % CIs were based on Hodges-Lehmann estimates of shift. Given the large number of comparisons and the post-hoc nature of the analyses, inferential statistics were not conducted in order to avoid issues of multiplicity.

Safety and tolerability were assessed within metabolic syndrome factor and insulin resistance subgroups. Clinical adverse experiences (AEs) were evaluated for all patients who were randomized and received at least one dose of study medication, and included any clinical AE, drug-related AE, serious AE, discontinuations due to an AE, and deaths. Laboratory AEs were evaluated for all subjects who had at least one post-baseline assessment, and included consecutive ≥ 3X the upper limit of normal (ULN) elevations in aspartate aminotransferase and/or alanine aminotransferase, ≥ 10X the ULN increases in creatine kinase with or without muscle symptoms, and a summary by system organ class and specific adverse experience term.

## Results

As reported previously, of the 1,143 subjects randomized to treatment, 658 subjects receiving atorvastatin monotherapy and 438 receiving ezetimibe/simvastatin completed the trial [14]. Baseline demographics within each metabolic syndrome factor and insulin resistance subgroup were generally similar to those found for the overall study population, [14] a predominantly white (75 %) and male (56 %) cohort with a mean age of 59 years (Table 1). Over half of the population was obese (body mass index ≥30 kg/m^2^), approximately 50-60 % was diabetic, and one third was classified as high CHD risk subjects with AVD. For each subgroup evaluation, baseline characteristics and lipid values were generally well balanced across the ezetimibe/simvastatin and atorvastatin treatment groups. As expected, baseline values for some parameters varied within a few subgroups due to the association between the parameter being evaluated and the subgroup category (i.e., the percentage of subjects with diabetes was much higher for the subgroup with fasting glucose levels ≥100 mg/dl [72-74 %] than for those with levels <100 mg/dl [10-11 %]; baseline triglyceride values were the highest in the subgroup of subjects with triglyceride levels ≥150 mg/dl). Variation in baseline values were noted for some categories (Black, Other) and subgroups (waist circumference < 40 in [<50 in for females], systolic blood pressure <130 mmHG [diastolic blood pressure <85 mmHg]) which was due in part to the small number of subjects being evaluated.

**Table 1:** Baseline demographics, lipids, lipoproteins, and hs-CRP

	Abdominal Obesity^a^ males (females)				TG				HDL-C males (females)				SB (DBP)				FG				HOMA-IR					
	<40 in	(<35in)	≥40 in	(≥35in)	<150 mg/dL		≥150 mg/dL		≥40 mg/dL (≥50 mg/dL)		<40 mg/dL (<50 mg/dL)		<130 mmHg (<85 mgHg)		≥130 mmHg (≥85 mmHg)		<100 mg/dL		≥100 mg/dL		<2.72		2.72 - 4.81		>4.81	
	All A (N = 94)	All E/S (N = 56)	All A (N = 586)	All E/S (N = 399)	All A (N = 212)	All E/S (N = 146)	All A (N = 474)	All E/S (N = 311)	All A (N = 287)	All E/S (N = 195)	All A (N = 399)	All E/S (N = 262)	All A (N = 56)	All E/S (N = 37)	All A (N = 630)	All E/S (N = 420)	All A (N = 196)	All E/S (N = 151)	All A (N = 490)	All E/S (N = 306)	All A (N = 232)	All E/S (N = 145)	All A (N = 225)	All E/S (N = 152)	All A (N = 218)	All E/S (N = 155)
Mean Age (SD)	59.2 (9.0)	62.4 (7.5)	58.7 (9.7)	59.3 (9.6)	60.2 (9.4)	60.3 (9.2)	58.1 (9.7)	59.3 (9.6)	60.5 (9.5)	60.7 (9.6)	57.5 (9.6)	58.8 (9.3)	53.5 (9.5)	51.6 (11.2)	59.2 (9.5)	60.3 (8.9)	59 (10.2)	58.8 (8.9)	58.7 (9.4)	60 (9.7)	60 (10.3)	60. 9 (8.6)	58.5 (8.7)	59.9 (9.9)	57. 6 (9.7)	58.2 (9.5)
Female, n(%)	24 (25.5)	9 (16.1)	281 (48.0)	180 (45.1)	106 (50.0)	68 (46.6)	201 (42.4)	123 (39.5)	107 (37.3)	66 (33.8)	200 (50.1)	125 (47.7)	31 (55.4)	13 (35.1)	276 (43.8)	178 (42.4)	78 (39.8)	51 (33.8)	229 (46.7)	140 (45.8)	102 (44.0)	57 (39.3)	100 (44.4)	67 (44.1)	100 (45.9)	66 (42.6)
White, n(%)	68 (72.3)	37 (66.1)	443 (75.6)	301 (75.4)	157 (74.1)	106 (72.6)	359 (75.7)	234 (75.2)	231 (80.5)	162 (83.1)	285 (71.4)	178 (67.9)	47 (83.9)	26 (70.3)	469 (74.4)	314 (74.8)	129 (65.8)	99 (65.6)	387 (79.0)	241 (78.8)	166 (71.6)	99 (68.3)	164 (72.9)	113 (74.3)	176 (80.7)	124 (80.0)
Black, n(%)	4 (4.3)	4 (7.1)	41 (7.0)	26 (6.5)	21 (9.9)	17 (11.6)	24 (5.1)	13 (4.2)	13 (4.5)	11 (5.6)	32 (8.0)	19 (7.3)	2 (3.6)	1 (2.7)	43 (6.8)	29 (6.9)	17 (8.7)	9 (6.0)	28 (5.7)	21 (6.9)	11 (4.7)	9 (6.2)	21 (9.3)	10 (6.6)	13 (6.0)	11 (7.1)
Other, n(%)	22 (23.4)	15 (26.8)	102 (17.4)	72 (18.0)	34 (16.0)	23 (15.8)	91 (19.2)	64 (20.6)	43 (15.0)	22 (11.3)	82 (20.6)	65 (24.8)	7 (12.5)	10 (27.0)	118 (18.7)	77 (18.3)	50 (25.5)	43 (28.5)	75 (15.3)	44 (14.4)	55 (23.7)	37 (25.5)	40 (17.8)	29 (19.1)	29 (13.3)	20 (12.9)
CHD n(%)	25 (26.6)	17 (30.4)	104 (17.7)	76 (19.0)	46 (21.7)	30 (20.5)	84 (17.7)	63 (20.3)	47 (16.4)	33 (16.9)	83 (20.8)	60 (22.9)	7 (12.5)	5 (13.5)	123 (19.5)	88 (21.0)	52 (26.5)	32 (21.2)	78 (15.9)	61 (19.9)	45 (19.4)	34 (23.4)	44 (19.6)	30 (19.7)	37 (17.0)	28 (18.1)
With AVD, n(%)	32 (34.0)	21 (37.5)	162 (27.6)	117 (29.3)	65 (30.7)	44 (30.1)	131 (27.6)	94 (30.2)	80 (27.9)	53 (27.2)	116 (29.1)	85 (32.4)	8 (14.3)	8 (21.6)	188 (29.8)	130 (31.0)	75 (38.3)	48 (31.8)	121 (24.7)	90 (29.4)	66 (28.4)	49 (33.8)	68 (30.2)	44 (28.9)	58 (26.6)	43 (27.7)
BMI ≥ 30 kg/m^2^, n(%)	16 (17.2)	7 (12.5)	387 (66.5)	267 (66.9)	133 (63.0)	89 (61.0)	276 (58.7)	186 (59.8)	164 (57.5)	112 (57.4)	245 (61.9)	163 (62.2)	31 (55.4)	23 (62.2)	378 (60.5)	252 (60.0)	96 (49.0)	80 (53.0)	313 (64.5)	195 (63.7)	91 (39.6)	60 (41.4)	141 (62.7)	102 (67.1)	170 (78.3)	110 (71.0)
Diabetes, n(%)	39 (41.5)	13 (23.2)	339 (57.8)	221 (55.4)	145 (68.4)	96 (65.8)	236 (49.8)	140 (45.0)	159 (55.4)	116 (59.5)	222 (55.6)	120 (45.8)	42 (75.0)	19 (51.4)	339 (53.8)	217 (51.7)	19 (9.7)	16 (10.6)	362 (73.9)	220 (71.9)	89 (38.4)	52 (35.9)	128 (56.9)	80 (52.6)	156 (71.6)	103 (66.5)
Metabolic Syndrome, n(%)	70 (74.5)	41 (73.2)	566 (96.6)	387 (97.0)	180 (85.3)	128 (88.3)	461 (97.3)	301 (96.8)	248 (86.7)	171 (87.7)	393 (98.5)	258 (98.9)	48 (85.7)	34 (91.9)	593 (94.3)	395 (94.3)	168 (85.7)	130 (86.7)	473 (96.7)	299 (97.7)	203 (87.9)	124 (86.1)	214 (95.1)	148 (97.4)	213 (97.7)	152 (98.1)
TG ≥ 150 mg/dL, n(%)	73 (77.7)	41 (73.2)	397 (67.7)	269 (67.4)	0	0	474 (100)	311 (100)	176 (61.3)	116 (59.5)	298 (74.7)	195 (74.4)	45 (80.4)	32 (86.5)	429 (68.1)	279 (66.4)	160 (81.6)	122 (80.8)	314 (64.1)	189 (61.8)	156 (67.2)	92 (63.4)	160 (71.1)	105 (69.1)	152 (69.7)	109 (70.3)
Low HDL-C, n(%)	58 (61.7)	36 (64.3)	337 (57.5)	224 (56.1)	101 (47.6)	67 (45.9)	298 (62.9)	195 (62.7)	0	0	399 (100)	262 (100)	47 (83.9)	31 (83.8)	352 (55.9)	231 (55.0)	122 (62.2)	101 (66.9)	277 (56.5)	161 (52.6)	122 (52.6)	85 (58.6)	128 (56.9)	88 (57.9)	143 (65.6)	87 (56.1)
Elevated BP, n(%)	85 (90.4)	53 (94.6)	539 (92.0)	365 (91.5)	201 (94.8)	141 (96.6)	429 (90.5)	279 (89.7)	278 (96.9)	189 (96.9)	352 (88.2)	231 (88.2)	0	0	630 (100)	420 (100)	183 (93.4)	142 (94.0)	447 (91.2)	278 (90.8)	212 (91.4)	134 (92.4)	205 (91.1)	139 (91.4)	204 (93.6)	142 (91.6)
FG ≥ 100 mg/dL, n(%)	55 (58.5)	30 (53.6)	431 (73.5)	275 (68.9)	176 (83.0)	117 (80.1)	314 (66.2)	189 (60.8)	213 (74.2)	145 (74.4)	277 (69.4)	161 (61.5)	43 (76.8)	28 (75.7)	447 (71.0)	278 (66.2)	0	0	490 (100)	306 (100)	124 (53.4)	70 (48.3)	165 (73.3)	102 (67.1)	192 (88.1)	132 (85.2)
LDL-C	138.2 (34.5)	139.1 (33.1)	140.6 (35.7)	135.1 (30)	131.1 (28.9)	133 (29.7)	144.4 (37.5)	136.6 (30.8)	145.7 (34.7)	138.5 (31.2)	136.4 (35.7)	133.2 (29.7)	140.6 (39.7)	131.9 (24.1)	140.2 (35.2)	135.7 (30.9)	148.2 (35.3)	139.7 (29.4)	137.1 (35.2)	133.4 (30.8)	146.7 (35.7)	142 (32.6)	139 (36.2)	134.8 (29.9)	135.6 (34.2)	130 (28)
HDL-C	41.5 (9.7)	42.5 (12.4)	43.3 (10.3)	43.2 (10.8)	46.3 (11.5)	46.8 (12.8)	41.6 (9.3)	41.3 (9.6)	50.3 (10.1)	50.7 (11.2)	37.8 (6.5)	37.6 (6.9)	41.4 (8.1)	37.3 (8.1)	43.2 (10.4)	43.6 (11.1)	41.9 (10.7)	40.4 (10.2)	43.5 (10.1)	44.4 (11.2)	44.2 (11.1)	42.9 (10.6)	43 (9.3)	43.7 (11.2)	41.8 (10.1)	43 (11.3)
non-HDL-C	177.4 (40.9)	179.8 (35.4)	181.1 (40.5)	174.6 (35.8)	156.9 (30.3)	157.9 (31.3)	191.3 (40.1)	183.2 (35)	181.7 (40.7)	173.5 (37.4)	179.9 (40.5)	176.1 (34.6)	182.7 (43.4)	181.6 (35.6)	180.5 (40.3)	174.4 (35.8)	191.8 (40.8)	182.4 (34.5)	176.3 (39.7)	171.5 (35.9)	183 (40.6)	178.5 (38)	181.3 (41.7)	174.8 (33.6)	178.1 (40)	171.9 (36.1)
TG^b^	171.5 (97.7)	188 (118.6)	186 (99.1)	177 (101.4)	123 (41.4)	121 (42.8)	215 (93.7)	215 (97.7)	162 (85.1)	157.5 (81.9)	200 (106)	199.8 (114.9)	194 (86.7)	217 (141.4)	181.5 (100)	175 (104.2)	201 (105.1)	201 (94.9)	175.8 (99.1)	161.5 (101.4)	167.5 (91.2)	174 (90.2)	183 (109.8)	177.5 (122.3)	196.3 (99.1)	183.5 (109.3)
Total C	218.9 (42.9)	222.3 (39.1)	224.3 (41.1)	218.1 (36.4)	203.2 (33.4)	204.8 (32.5)	232.8 (41.5)	224.9 (37)	232 (40.1)	224.2 (37.7)	217.6 (41.4)	214.2 (35.7)	223.3 (44.1)	218.9 (35)	223.6 (41.2)	218.4 (37)	233.6 (43.3)	222.8 (36.1)	219.7 (40)	216.3 (37)	227 (41.3)	222.4 (41.2)	224.4 (41.9)	218.5 (33.2)	219.9 (41.6)	214.8 (36.1)
Apo B	134.3 (29.2)	137.6 (25.2)	137.3 (30.1)	133.4 (26.5)	121.6 (23.2)	122.7 (22.1)	143.8 (30.3)	138.9 (26.9)	136.5 (29.5)	131.5 (25.9)	137.2 (30.5)	135.2 (26.8)	141.5 (33.2)	138.5 (26.1)	136.5 (29.8)	133.3 (26.5)	144.1 (30.3)	139.8 (25.4)	134 (29.6)	130.7 (26.6)	138.2 (29.5)	138 (27.8)	137.1 (32.3)	132.7 (24.7)	135.5 (28.8)	130.3 (26.5)
Apo AI	140.4 (24.6)	141.5 (27.7)	144.8 (24.3)	144.8 (25.5)	145 (26.3)	146.3 (27.9)	143.7 (23.6)	143.4 (24.6)	157.7 (24.8)	160.7 (26.2)	134.3 (18.8)	132.7 (17.9)	141 (20.1)	136.8 (25.6)	144.4 (24.8)	145 (25.7)	142.1 (26.6)	138.4 (24.7)	144.9 (23.5)	147.2 (25.8)	143.6 (26.5)	142.3 (24.4)	144.7 (22.9)	146.3 (25.4)	143.9 (23.5)	144.5 (27.4)
hs-CRP^b^	1.8 (2.5)	2(3)	3.3 (4.6)	3.2 (4.7)	3 (5.4)	2.8 (4.4)	3 (4.1)	3.1 (4.2)	2.7 (3.7)	2.4 (3.3)	3.3 (4.8)	3.5 (5.8)	2.3 (2.8)	4.2 (4)	3 (4.6)	2.9 (4.6)	2.9 (3.8)	2.5 (3.1)	3 (4.7)	3.5 (5.3)	2.3 (3.6)	2.3 (3.9)	3 (4)	3.1 (4)	4 (5.3)	3.5 (6)

Apo = apolipoprotein; All A = data for all atorvastatin doses (10/20/40 mg) combined; Apo = apolipoprotein; BMI = body mass index; BP = blood pressure; All E/S = data for all ezetimibe/simvastatin doses (10/20 mg, 10/40 mg) combined; FG = fasting glucose; HDL-C = high density lipoprotein cholesterol; hsCRP = high-sensitivty C-reactive protein, LDL-C = low density lipoprotein cholesterol, n = number of patients in subgroup (numbers vary slightly between parameters), TG = triglycerides; SD = standard devation ^a^metabolic syndrome defined as ≥3 of the following 5 characteristics; abdominal obesity (waist circumference ≥40 inches (males) or ≥35 inches (females)), TG ≥150 mg/dL, low HDL-C (<40 mg/dL (males) or <50 mg/dL (females)), elevated BP (≥130/85 mmHg or on antihypertensive medication or diagnosis of hypertension based on medical history), FG ≥100 mg/dL. ^b^robust SD

**Fig. 1:**
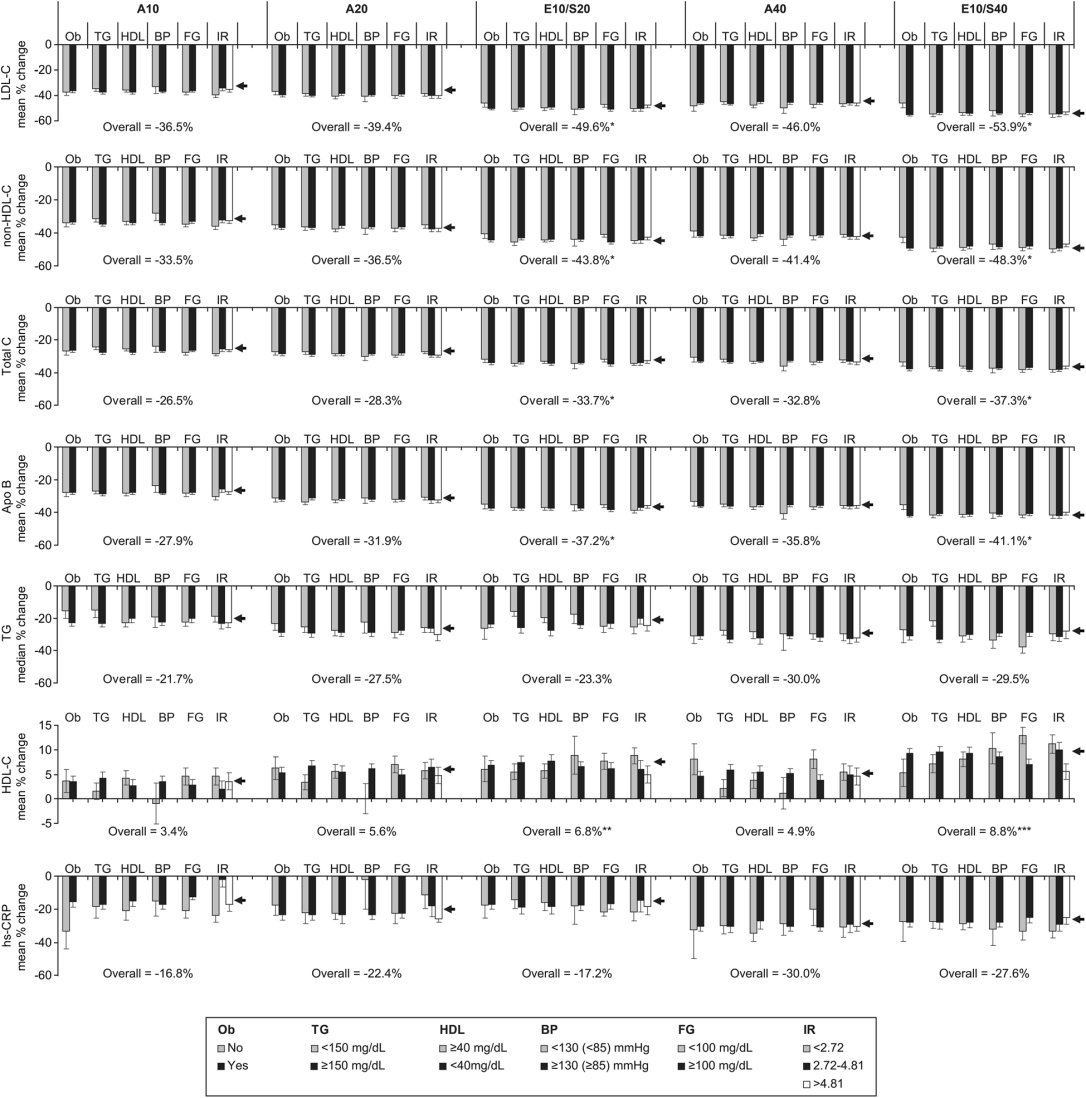
Percent change from baseline in lipid, lipoprotein, and hs-CRP by treatment and metabolic syndrome/IR subgroup. Error bars = standard error. Arrows indicate percent change from baseline reported for the overall study population [13], with associated value given below the measured parameter for each treatment group. A10/20/40 = atorvastatin 10/20/40 mg; Apo = apolipoprotein; BP = blood pressure [systolic blood pressure (diastolic blood pressure)]; E10/S20(40) = ezetimibe 10 mg/simvastatin 20(40) mg; FG = fasting glucose; HDL-C = high density lipoprotein cholesterol; IR = Homeostasis Model Assessment of Insulin Resistance tertiles; hs-CRP = high-sensitivity C-reactive protein, LDL-C = low- density lipoprotein cholesterol; Ob = abdominal obesity (waist circumference ≥40 inches for males or ≥35 inches for females); TG = triglycerides

Ezetimibe/simvastatin (10/20 mg, 10/40 mg) and atorvastatin (10, 20, 40 mg) therapy produced relatively consistent lipid-altering effects across all metabolic syndrome factor and insulin resistant subgroups, which were similar in magnitude to those previously reported for the overall study population (Fig. 1) [14]. All treatments produced significant reductions from baseline in LDL-C, non-HDL-C, total cholesterol, apo B, triglycerides, and lipid/lipoprotein ratios (total cholesterol:HDL-C, LDL-C:HDL-C, apo B:apo AI, non-HDL-C:HDL-C, data not shown) for all subgroups. VLDL-C (data not shown) and hs-CRP changes from baseline were also significant for most subgroup evaluations and consistent with the changes observed for the full cohort. Increases in HDL-C were observed for all subgroups except two (subjects receiving atorvastatin 10 mg and 20 mg with systolic blood pressure <130 mm Hg [diastolic <85 mm Hg], n = 11 and 21, respectively) and most changes were nominally significant. Elevations in apo AI were smaller in magnitude than HDL-C, and most were not significantly different from baseline (data not shown). When comparing ezetimibe/simvastatin 10/20 mg with atorvastatin 10 mg or 20 mg, and ezetimibe/simvastatin 10/40 mg with atorvastatin 40 mg, treatment with ezetimibe/simvastatin produced generally greater percent reductions from baseline in LDL-C, non-HDL-C, Apo B, total cholesterol, and lipoprotein ratios (data not shown) for all but four subgroup comparisons, with between treatment differences ranging from 0.4 to 27.6 % (Table 2); atorvastatin 40 mg produced greater reductions than ezetimibe/simvastatin 10/40 mg for LDL-C by 2.0 %, LDL-C:HDL-C by 4.5 % and apoB:apoA1 by 1.3 % for subjects with waist circumferences <40/35 inches, and similar reductions in apoB (0.1 %) for subjects with blood pressure <130/85 mm Hg. Assessment of the associated 95 % CIs suggest that the lipid-lowering effects of ezetimibe/simvastatin were greater than atorvastatin at the doses compared for most subgroups (Table 2), which is consistent with the significantly greater differences previously reported for the entire cohort (Fig. 1) [14]. When compared with atorvastatin, ezetimibe/simvastatin produced numerically larger percent increases in HDL-C and apo AI for all but three subgroups (non-obese, HDL-C ≥40 mg/dl, and HOMA-IR 2.72-4.81 (Table 2). The percent changes from baseline in VLDL-C (not shown), triglycerides, and hs-CRP were similar for the majority of ezetimibe/simvastatin and atorvastatin comparisons and were consistent with the similar treatment effects seen for the overall study.

**Table 2:** Percent differences within metabolic syndrome and insulin resistance subgroups in LDL-C, total cholesterol, HDL-C and triglycerides

	Abdominal Obesity^a^		TG		HDL-C		SBP (DBP)		FG		HOMA-IR		
	No	Yes	<150 mg/dL	≥150 mg/dL	≥40 mg/dL	<40 mg/dL	<130 (85) mmHg	≥130 (85) mmHg	<100 mg/dL	≥100 mg/dL	<2.72	2.72-4.81	>4.81
	n = 20-36	n = 177-196	n = 62-77	n = 142-155	n = 82-99	n = 119-135	n = 11-21	n = 194-205	n = 52-75	n = 144-165	n = 57-81	n = 68-78	(n = 69-79
LDL-C, mg/dL (95 % CI)													
E/S20 - A10^b^	−8.6 (−17.3, 0.1)	−13.9 (−17.6, −0.3)*	−15.8 (−21.7, −10.0)*	−11.8 (−15.9, −7.7)*	−14.3 (−19.3, −9.2)*	−12.2 (−16.7, −7.8)*	−17.8 (−31.8, −3.8)*	−12.9 (−16.3, −9.4)*	−9.5 (−15.4, −3.6)*	−14.9 (−19.0, −10.9)*	−11.1 (−16.7, −5.4)*	−15.9 (−21.8, −10.0)*	−12.3 (−18.2, −6.5)*
E/S20 - A20^b^	−9.1 (−17.8, −0.4)*	−10.4 (−14.1, −6.8)*	−12.0 (−17.7, −6.3)*	−9.3 (−13.4, −5.2)*	−9.1 (−14.1, −4.1)*	−11.1 (−15.6, −6.6)*	−9.8 (−21.8, 2.2)	−10.3 (−13.8,-6.8)*	−6.8 (−12.7, −0.9)*	−11.9 (−16.0, −7.9)*	−12.3 (−17.9, −6.7)*	−10.3 (−16.2, −4.4)*	−7.7 (−13.5, −1.8)*
E/S40 - A40^b^	2.0 (−8.4, 12.4)	−9.3 (−12.8, −5.8)*	−9.8 (−16.0, −3.6)*	−7.2 (−11.2,-3.2)*	−5.90 (−11.3, −0.5)*	−9.2 (−13.4, −4.9)*	−2.4 (−13.5, 8.8)	−8.5 (−12.0, −5.0)*	−7.6 (−14.0,-1.1)*	−8.0 (−11.9,-4.1)*	−8.1 (−14.2, −2.0)*	−8.9 (−14.5, −3.2)*	−7.1 (−12.9, −1.4)*
TC, mg/dL (95 % CI)													
E/S20 - A10^b^	−4.8 (−11.2, 1.5)	−7.7 (−10.3, −5.1)*	−10.2 (−14.4, −5.9)*	−5.8 (−8.8, −2.9)*	−7.6 (−11.2, −3.9)*	−7.0 (−10.2, −3.8)*	−10.6 (−20.8, −0.5)*	−7.0 (−9.5, −4.5)*	−4.3 (−8.5, 0.0)	−8.7 (−11.6, −5.7)*	−6.1 (−10.2, −2.0)*	−8.4 (−12.6, −4.1)*	−7.1 (−11.4, −2.9)*
E/S20 - A20^b^	−4.8 (−11.0, 1.5)	−5.5 (−8.1, −2.8)*	−7.1 (−11.2, −3.0)*	−4.4 (−7.4, −1.4)*	−4.6 (−8.2,-1.0)*	−6.0 (−9.2, −2.7)*	−4.6 (−13.3, 4.2)	−5.5 (−8.0, −2.9)*	−2.8 (−7.1, 1.5)	−6.6 (−9.6, −3.7)*	−7.4 (−11.4, −3.3)*	−4.8 (−9.1, −0.5)*	−3.7 (−8.0, 0.6)
E/S40 - A40^b^	−3.0 (−10.6, 4.6)	−4.8 (−7.3, −2.2)*	−4.3 (−8.8, 0.2)	−4.5 (−7.4, −1.6)*	−3.1 (−7.0, 0.9)	−5.3 (−8.4, −2.2)*	−1.0 (−9.0, 7.0)	−4.8 (−7.3, −2.2)*	−4.9 (−9.6, −0.2)*	−4.1 (−7.0, −1.3)*	−5.8 (−10.2,-1.3)*	−4.8 (−8.9, −0.6)*	−2.6 (−6.8, 1.5)
HDL-C, mg/dL (95 % CI)													
E/S20 - A10^b^	2.4 (−4.5, 9.3)	3.3 (0.4, 6.2)*	3.9 (−0.7, 8.5)	3.2 (0.0, 6.5)	1.3 (−2.6, 5.3)	5.0 (1.4, 8.5)*	9.8 (−1.3, 20.8)	3.0 (0.3, 5.7)*	3.2 (−1.4, 7.8)	3.4 (0.2, 6.6)*	4.2 (−0.2, 8.7)	4.1 (−0.5, 8.8)	1.4 (−3.2, 6.0)
E/S20 -A20^b^	−0.2 (−7.0, 6.7)	1.6 (−1.3, 4.5)	2.0 (−2.5, 6.5)	0.8 (−2.5, 4.0)	0.0 (−3.9, 3.9)	2.2 (−1.4, 5.7)	8.9 (−0.6, 18.4)	0.4 (−2.3, 3.2)	0.8 (−3.9, 5.4)	1.3 (−1.9, 4.5)	3.0 (−1.4, 7.4)	−0.3 (−5.0, 4.4)	0.2 (−4.4, 4.9)
E/S40 - A40^b^	−2.8 (−11.0, 5.5)	4.7 (1.9, 7.5)*	5.0 (0.1, 9.9)*	3.6 (0.5, 6.7)*	4.3 (0.1, 8.6)*	3.7 (0.4, 7.1)*	9.1 (0.3, 17.9)*	3.5 (0.7, 6.2)*	4.8 (−0.3, 9.9)	3.2 (0.1, 6.3)*	5.6 (0.8, 10.5)*	4.9 (0.4, 9.4)*	1.1 (−3.5, 5.6)
TG, mg/dL (95 % CI)													
E/S20 - A10^b^	−7.4 (−20.3, 6.4)	0.1 (−4.7, 4.6)	−0.9 (−9.0, 6.2)	−1.5 (−6.8, 3.5)	1.8 (−5.2, 8.3)	−3.1 (−9.1, 2.4)	−2.7 (−20.8, 11.1)	−0.8 (−5.4, 3.6)	−0.3 (−7.9, 7.7)	−1.3 (−6.8, 3.9)	−3.9 (−11.7, 3.9)	1.4 (−6.1, 8.8)	−0.3 (−8.1, 6.9)
E/S20 - A20^b^	−0.8 (−12.9, 11.5)	3.5 (−1.3, 8.2)	7.3 (−0.2, 14.8)	0.3 (−5.0, 5.7)	4.3 (−2.3, 11.1)	1.7 (−4.1, 7.3)	2.1 (−11.1, 16.2)	2.9 (−1.7, 7.5)	2.4 (−4.7, 10.8)	2.7 (−2.5, 8.3)	−0.1 (−8.0, 8.2)	4.2 (−3.5, 12.2)	3.6 (−4.0, 10.5)
E/S40 - A40^b^	−0.3 (−19.6, 15.4)	−0.4 (−4.9, 4.1)	2.2 (−6.6, 10.9)	−1.4 (−6.4, 3.5)	−1.6 (−8.8, 5.1)	0.4 (−5.3, 5.8)	−2.7 (−18.5, 11.5)	−0.1 (−4.7, 4.5)	−6.1 (−13.9, 2.6)	1.9 (−3.4, 6.9)	−4.0 (−12.7, 4.9)	−1.2 (−8.2, 5.7)	4.8 (−3.3, 12.3)

A10/20/40 = atorvastatin 10/20/40 mg; E = ezetimibe 10 mg; FG = fasting glucose; HDL-C = high density lipoprotein cholesterol; HOMA-IR = Homeostasis Model Assessment of Insulin Resistance; LDL-C = low density lipoprotein cholesterol, n = number of patients in subgroup (some numbers vary slightly between parameters), S20/40 = simvastatin 20/40 mg; SBP (DBP) = systolic blood pressure (diastolic blood pressure); SD = standard deviation; TG = triglycerides

*Confidence intervals of difference parameters do not contain 0 ^a^abdominal obesity = waist circumference ≥40 inches for males or ≥35 inches for females ^b^between-treatment differences (E+S minus A) based on ANOVA model with terms for subgroup (baseline abdominal obesity, TG, HDL-C, blood pressure, fasting glucose, and HOMA-IR, based on subgroup being analyzed), baseline stratum, treatment group, and the interaction of treatment group and subgroup Values in parentheses = 95% confidence intervals

All doses of ezetimibe/simvastatin and atorvastatin were generally safe and well tolerated, with an incidence of one or more adverse experiences (11.3 to 23.2 %), drug-related adverse experiences (1.4 to 5.7 %), and serious adverse experiences (0 to 1.8 %) that was generally similar across all subgroups (Table 3) and consistent with the primary study results [14]. The statistical significance of differences between subgroups and between treatments within each subgroup was not evaluated due to the *post hoc* nature of these analyses.

**Table 3:** Adverse experience summary of pooled treatment groups

Adverse Experience	Obesity				TG				HDL-C				SB/ DBP				FG				HOMA-IR					
	males/females								males/females																	
n (%)																										
	<40 in/<35in)		≥40 in/≥35in)		<150 mg/dL		≥150 mg/dL		≥40 mg/dL/ ≥50 mg/dL		<40 mg/dL/ <50 mg/dL		<130 mmHg/ <85 mgHg		≥130 mmHg/ ≥ 85 mmHg		<100 mg/dL		≥100 mg/dL		<2.72		2.72 - 4.81		>4.81	
	All A	E/All S	All A	E/All S	All A	E/All S	All A	E/All S	All A	E/All S	All A	E/All S	All A	E/All S	All A	E/All S	All A	E/All S	All A	E/All S	All A	E/All S	All A	E/All S	All A	E/All S
	n = 94	n = 56	n = 578	n = 392	n = 209	n = 144	n = 469	n = 306	n = 282	n = 190	n = 396	n = 260	n = 56	n = 36	n = 622	n = 414	n = 192	n = 147	n = 486	n = 303	n = 229	n = 142	n = 222	n = 151	n = 218	n = 154
With one or more AE	14 (14.9)	12 (21.4)	120 (20.8)	64 (16.3)	40 (19.1)	21 (14.6)	94 (20.0)	55 (18.0)	57 (20.2)	35 (18.4)	77 (19.4)	41 (15.8)	8 (14.3)	6 (16.7)	126 (20.3)	70 (16.9)	39 (20.3)	26 (17.7)	95 (19.5)	50 (16.5)	47 (20.5)	33 (23.2)	43 (19.4)	17 (11.3)	40 (18.3)	26 (16.9)
With drug-related AE^a^	2 (2.1)	2 (3.6)	24 (4.2)	13 (3.3)	3 (1.4)	4 (2.8)	23 (4.9)	11 (3.6)	11 (3.9)	7 (3.7)	15 (3.8)	8 (3.1)	2 (3.6)	1 (2.8)	24 (3.9)	14 (3.4)	11 (5.7)	5 (3.4)	15 (3.1)	10 (3.3)	4 (1.7)	5 (3.5)	10 (4.5)	3 .0 (2.0)	11 (5.0)	7 (4.5)
With serious AE	1 (1.1)	0 (0)	8 (1.4)	1 (0.3)	2 (1.0)	1 (0.7)	7 (1.5)	0 (0)	4 (1.4)	0 (0)	5 (1.3)	1 (0.4)	1 (1.8)	0 (0)	8 (1.3)	1 (0.2)	3 (1.6)	1 (0.7)	6 (1.2)	0 (0)	3 (1.3)	0 (0)	3 (1.4)	1 (0.7)	2 (0.9)	0 (0)
With serious drug-related AE	0 (0)	0 (0)	1 (0.2)	0 (0)	0 (0)	0 (0)	1 (0.2)	0 (0)	1 (0.4)	0 (0)	0 (0)	0 (0)	0 (0)	0 (0)	1 (0.2)	0 (0)	0 (0)	0 (0)	1 (0.2)	0 (0)	0 (0)	0 (0)	1 (0.5)	0 (0)	0 (0)	0 (0)
Discontinued due to AE	1 (1.1)	0 (0)	11 (1.9)	6 (1.5)	1 (0.5)	2 (1.4)	11 (2.3)	4 (1.3)	4 (1.4)	3 (1.6)	8 (2.0)	3 (1.2)	1 (1.8)	0 (0)	11 (1.8)	6 (1.4)	3 (1.6)	1 (0.7)	9 (1.9)	5 (1.7)	2 (0.9)	1 (0.7)	4 (1.8)	1 (0.7)	4 (1.8)	4 (2.6)
drug-related	0 (0)	0 (0)	7 (1.2)	4 (1.0)	0 (0)	1 (0.7)	7 (1.5)	3 (1.0)	2 (0.7)	3 (1.6)	5 (1.3)	1 (0.4)	1 (1.8)	0 (0)	6 (1.0)	4 (1.0)	2 (1.0)	1 (0.7)	5 (1.0)	3 (1.1)	1 (0.4)	0 (0)	2 (0.9)	1 (0.7)	3 (1.4)	3 (1.9)
serious	0 (0)	0 (0)	2 (0.3)	0 (0)	0 (0)	0 (0)	2 (0.4)	0 (0)	2 (0.7)	0 (0)	0 (0)	0 (0)	0 (0)	0 (0)	2 (0.3)	0 (0)	0 (0)	0 (0)	2 (0.4)	0 (0)	0 (0)	0 (0)	1 (0.5)	0 (0)	1 (0.5)	0 (0)
serious drug-related	0 (0)	0 (0)	1 (0.2)	0 (0)	0 (0)	0 (0)	1 (0.2)	0 (0)	1 (0.4)	0 (0)	0 (0)	0 (0)	0 (0)	0 (0)	1 (0.2)	0 (0)	0 (0)	0 (0)	1 (0.2)	0 (0)	0 (0)	0 (0)	1 (0.5)	0 (0)	0 (0)	0 (0)
Deaths	0 (0)	0 (0)	0 (0)	0 (0)	0 (0)	0 (0)	0 (0)	0 (0)	0 (0)	0 (0)	0 (0)	0 (0)	0 (0)	0 (0)	0 (0)	0 (0)	0 (0)	0 (0)	0 (0)	0 (0)	0 (0)	0 (0)	0 (0)	0 (0)	0 (0)	0 (0)
Pre-specified^b^ m/n (%)																										
ALT or AST ≥ 3xULN, consecutive^c^	0/92	1/55 (1.8)	2/559 (0.4)	5/385 (1.3)	0/203	1/142 (0.7)	2/454 (0.4)	5/300 (1.7)	2/273 (0.7)	5/186 (2.7)	0/384	1/256 (0.4)	0/53	1/35 (2.9)	2/604 (0.3)	5/407 (1.2)	1/188 (0.5)	2/143 (1.4)	1/469 (0.2)	4/299 (1.3)	0/222	3/141 (2.1)	2/216 (0.9)	1/147 0.7)	0/211	2/151 (1.3)
ALT ≥ 3xULN, consecutive^c^	0/92	0/55	2/558 (0.4)	1/385 (0.3)	0/203	0/142	2/453 (0.4)	1/300 (0.3)	2/272 (0.7)	0/186	0/384	1/256 (0.4)	0/53	0/35	2/603 (0.3)	1/407 (0.2)	1/188 (0.5)	1/143 (0.7)	1/468 ( 0.2)	0/299	0/222	1/141 (0.7)	2/216 (0.9)	0/147	0/210	0/151
AST ≥ 3xULN, consecutive^c^	0/92	1/55 (1.8)	1/559 (0.2)	4/385 (1.0)	0/203	1/142 (0.7)	1/454 (0.2)	4/300 (1.3)	1/273 (0.4)	5/186 (2.7)	0/384	0/256	0/53	1/35 (2.9)	1/604 (0.2)	4/407 (1.0)	0/188	1/143 (0.7)	1/469 (0.2)	4/299 (1.3)	0/222	2/141 (1.4)	1/216 (0.5)	1/147 (0.7)	0/211	2/151 (1.3)
CK ≥ 10xULN	0/92	1/55 (1.8)	0/559	0/385	0/203	0/142	0/454	1/300 (0.3)	0/273	1/186 (0.5)	0/384	0/256	0/53	0/35	0/604	1/407 (0.2)	0/188	1/143 (0.7)	0/469	0/299	0/222	1/141 (0.7)	0/216	0/147	0/211	0/151
CK ≥ 10xULN with muscle symptoms	0/92	0/55	0/559	0/385	0/203	0/142	0/454	0/300	0/273	0/186	0/384	0/256	0/53	0/35	0/604	0/407	0/188	0/143	0/469	0/299	0/222	0/141	0/216	0/147	0/211	0/151
CK ≥ 10xULN with drug-related muscle symptoms	0/92	0/55	0/559	0/385	0/203	0/142	0/454	0/300	0/273	0/186	0/384	0/256	0/53	0/35	0/604	0/407	0/188	0/143	0/469	0/299	0/222	0/141	0/216	0/147	0/211	0/151
Hepatitis-related AEs	0/94	0/56	0/578	0/392	0/209	0/144	0/469	0/306	0/282	0/190	0/396	0/260	0/56	0/36	0/622	0/414	0/192	0/147	0/486	0/303	0/229	0/142	0/222	0/151	0/218	0/154
Gallbladder-related AEs	0/94	0/56	1/578 ( 0.2)	0/392	0/209	0/144	1/469 ( 0.2)	0/306	1/282 (0.4)	0/190	0/396	0/260	0/56	0/36	1/622 (0.2)	0/414	0/192	0/147	1/486 (0.2)	0/303	0/229	0/142	1/222 (0.5)	0/151	0/218	0/154
Gastrointestinal-related AEs	4/94 (4.3)	4/56 (7.1)	24/578 (4.2)	21/392 (5.4)	8/209 (3.8)	8/144 (5.6)	20/469 (4.3)	17/306 (5.6)	10/282 (3.5)	14/190 (7.4)	18/396 (4.5)	11/260 (4.2)	1/56 (1.8)	1/36 (2.8)	27/622 (4.3)	24/414 (5.8)	10/192 (5.2)	11/147 (7.5)	18/486 (3.7)	14/303 ( 4.6)	10/229 (4.4)	12/142 (8.5)	12/222 (5.4)	5/151 (3.3)	6/218 (2.8)	8/154 (5.2)
Allergic reaction or rash AEs	1/94 (1.1)	1/56 (1.8)	5/578 (0.9)	4/392 (1.0)	2/209 (1.0)	1/144 (0.7)	4/469 (0.9)	4/306 (1.3)	2/282 (0.7)	5/190 (2.6)	4/396 (1.0)	0/260	0/56	0/56	6/622 (1.0)	5/414 (1.2)	3/192 (1.6)	0/147	3/486 (0.6)	5/303 (1.7)	1/229 (0.4)	1/142 (0.7)	2/222 (0.9)	2/151 (1.3)	3/218 (1.4)	2/154 (1.3)

Although a patient may have had two or more clinical adverse experiences the patient is counted only once in a category. The same patient may appear in different categories

E = Ezetimibe 10 mg; All Atorva = Atorvastatin(10,20,40 mg) pooled across all doses; E/All Simva = Ezetimibe/Simvastatin(10/20, 10/40 mg) pooled across all doses; n = Number of randomized and treated patients in each treatment group

^a^Determined by the investigator to be possibly, probably, or definitely related to the drug

^b^For laboratory safety (ALT, AST, CK), patients must have taken at least one dose of study medication and have at least one postbaseline measurement within 14 days of the last dose of study therapy to be included in the analysis

% = m/n x 100 = (number of patients within the Tier 1 adverse event category / number of treated patients [with one or more laboratory tests postbaseline, if parameter is a laboratory parameter]) × 100

^c^This category includes those patients with (a) two consecutive measurements ≥3xULN, (b) a single, last measurement ≥3xULN, or (c) a measurement ≥3xULN followed by a measurement <3xULN that was taken more than 2 days after the last dose of study medication

## Discussion

This *post hoc* study of subjects with hypercholesterolemia, metabolic syndrome, and moderately high or high CHD risk was designed to evaluate the effect of individual metabolic syndrome factors and insulin resistance on the lipid-lowering efficacy of ezetimibe/simvastatin (10/20 mg, 10/40 mg) and atorvastatin (10, 20, 40 mg). Results showed a consistent treatment effect for both ezetimibe/simvastatin and atorvastatin when comparing subjects with to those without the individual metabolic syndrome factors of abdominal obesity (based on waist circumference), elevated triglycerides, low HDL-C, high blood pressure, and fasting glucose ≥100 mg/dl; or within low, middle, or high tertile subgroups of insulin resistance as measured by HOMA-IR. The magnitude of lipid-altering effects produced by each treatment regimen was generally similar across all subgroups, suggesting that ezetimibe/simvastatin and atorvastatin treatment effects are not substantively influenced by these factors. Ezetimibe/simvastatin produced greater percent reductions in LDL-C, non-HDL-C, apo B, total cholesterol, and lipid/lipoprotein ratios than atorvastatin for most subgroups and dose comparisons. These effects were similar to the results reported for the overall study cohort [14] and consistent with three other studies designed to evaluate ezetimibe/simvastatin and atorvastatin efficacy in subjects at moderately high or high CHD risk with hypercholesterolemia (VYVA) [17], with diabetes mellitus and hypercholesterolemia (VYTAL) [18], or with advanced age (≥65 years) and hypercholesterolemia (VYTELD) [19]. In addition, a *post hoc* multivariate analysis demonstrated that ezetimibe/simvastatin treatment was consistently more effective than atorvastatin treatment in at-risk patients with the MetS in patients ≥65 years, in those with abdominal obesity, and with lower baseline hs-CRP at the specified dose comparisons (VYMET) [20].

The altered regulation of lipid metabolism found in patients with metabolic syndrome, including low HDL-C, high triglycerides, and elevated concentrations of small-dense LDL (sdLDL) particles, seems to play a major role in elevating cardiovascular disease risk [13]. In patients with metabolic syndrome, high triglyceride content in the liver is associated with the increased hepatic secretion of triglyceride-rich VLDL. Cholesteryl ester transfer protein (CETP) mediates the exchange of cholesteryl ester for triglyceride between triglyceride-rich lipoproteins and HDL, resulting in HDL with increased susceptibility to hepatic lipase, generation of smaller HDL particles, and accelerated HDL catabolism. A similar CETP-mediated process is responsible for the formation of sdLDL; however, these particles have a slower catabolic rate and increased atherogenicity [21]. Atorvastatin has been shown to produce a dose-dependent reduction in sdLDL with a concomitant shift from the more atherogenic small-dense to larger, less dense LDL particles for subjects with hypertriglyceridemia [22] and other forms of dyslipidemia [23]. Ezetimibe alone or in combination with statins has also been shown to reduce levels of sdLDL [24–26]. While lipid-lowering therapy has a demonstrated effect on lowering sdLDL, effects of individual metabolic syndrome factors or insulin resistance on sdLDL kinetics during treatment are unknown and require further investigation.

As seen for the overall study, all treatments were generally well tolerated and clinical adverse experiences were similar across all metabolic syndrome factor and insulin resistance subgroups. Because the subgroup sizes were relatively small and the event rates were low overall, it is difficult to generalize these results for subgroups of patients with individual components of the metabolic syndrome or insulin resistance.

As a *post hoc* analysis, results from this study have several limitations and should be interpreted with appropriate caution. Since many of the subgroups were limited in size when compared with the entire cohort and multiple comparisons were made, results may not truly be representative of a given subpopulation. This study was also not designed to have adequate power to determine the statistical significance for between-treatment differences in subgroups. These findings, however, are consistent with several prior studies showing the greater lipid-lowering efficacy of ezetimibe/simvastatin therapy versus statin monotherapy in patients with diabetes or metabolic syndrome [27–30]. The short duration of this study precludes evaluation of long-term treatment efficacy or safety.

In summary, results from this study suggest that ezetimibe/simvastatin and atorvastatin efficacy is generally well maintained across metabolic syndrome factor and insulin resistance subgroups. Ezetimibe/simvastatin provided greater lipid-altering benefits for most key parameters than atorvastatin at the doses compared, which may be of interest to clinicians when evaluating treatment options for patients with metabolic syndrome and moderately high to high CHD risk. Additional studies will be needed to determine the final clinical significance of these results.

## Abbreviations

ACC: American College of Cardiology
AHA: American Heart Association
AE: Adverse experience
ANOVA: Analysis of variance
Apo: Apolipoprotien
AVD: Atherosclerotic vascular disease
CETP: Cholesteryl ester transfer protein
CI: Confidence interval
CHD: Coronary heart disease
HDL-C: High-density lipoprotein cholesterol
HOMA-IR: Homeostasis Model Assessment of Insulin Resistance
hs-CRP: High-sensitivity C-reactive protein
LDL-C: Low-density lipoprotein cholesterol
sdLDL: Small-dense low-density lipoprotien
ULN: Upper limit of normal
VLDL: Very-low density lipoprotein
VYMET: Vytorin in Metabolic Syndrome

## References

[1] Grundy SM, Cleeman JI, Daniels SR, Donato KA, Eckel RH, Franklin BA, et al. Diagnosis and management of the metabolic syndrome: an American Heart Association/National Heart, Lung, and Blood Institute Scientific Statement. Circulation. 2005;112:2735–52. 10.1161/CIRCULATIONAHA.105.169404 16157765

[2] Mottillo S, Filion KB, Genest J, Joseph L, Pilote L, Poirier P, et al. The metabolic syndrome and cardiovascular risk a systematic review and meta-analysis. J Am Coll Cardiol. 2010;56:1113–32. 10.1016/j.jacc.2010.05.034 20863953

[3] Cornier MA, Dabelea D, Hernandez TL, Lindstrom RC, Steig AJ, Stob NR, et al. The Metabolic Syndrome. Endocr Rev. 2008;29:777–822. 10.1210/er.2008-0024 PMC5393149 18971485

[4] Grundy SM (2007). Controversy in clinical endocrinology - Metabolic syndrome: A multiplex cardiovascular risk factor. J Clin Endocrinol Metab.

[5] Grundy SM (2008). Metabolic syndrome pandemic. Arterioscler Thromb Vasc Biol.

[6] Ervin RB. Prevalence of metabolic syndrome among adults 20 years of age and over, by sex, age, race and ethnicity, and body mass index: United States, 2003–2006. Natl Health Stat Report 2009;1–7 19634296

[7] Alberti KG, Eckel RH, Grundy SM, Zimmet PZ, Cleeman JI, Donato KA, et al. Harmonizing the metabolic syndrome: a joint interim statement of the International Diabetes Federation Task Force on Epidemiology and Prevention; National Heart, Lung, and Blood Institute; American Heart Association; World Heart Federation; International Atherosclerosis Society; and International Association for the Study of Obesity. Circulation. 2009;120:1640–5. 10.1161/CIRCULATIONAHA.109.192644 19805654

[8] Third Report of the National Cholesterol Education Program (NCEP) Expert Panel on Detection, Evaluation, and Treatment of High Blood Cholesterol in Adults (Adult Treatment Panel III) final report. Circulation 2002; 106: 3143–3421. 12485966

[9] Brunzell JD, Davidson M, Furberg CD, Goldberg RB, Howard BV, Stein JH, et al. Lipoprotein management in patients with cardiometabolic risk: consensus statement from the American Diabetes Association and the American College of Cardiology Foundation. Diabetes Care. 2008;31:811–22. 10.2337/dc08-9018 18375431

[10] Perk J, De Backer G, Gohlke H, Graham I, Reiner Z, Verschuren M, et al. European Guidelines on cardiovascular disease prevention in clinical practice (version 2012). The Fifth Joint Task Force of the European Society of Cardiology and Other Societies on Cardiovascular Disease Prevention in Clinical Practice (constituted by representatives of nine societies and by invited experts). Eur Heart J. 2012;33:1635–701. 10.1093/eurheartj/ehs092 22555213

[11] Genest J, McPherson R, Frohlich J, Anderson T, Campbell N, Carpentier A, et al. 2009 Canadian Cardiovascular Society/Canadian guidelines for the diagnosis and treatment of dyslipidemia and prevention of cardiovascular disease in the adult - 2009 recommendations. Can J Cardiol. 2009;25:567–79.

[12] Stone NJ, Robinson J, Lichtenstein AH, Bairey Merz CN, Blum CB, Eckel RH, et al. 2013 ACC/AHA Guideline on the Treatment of Blood Cholesterol to Reduce Atherosclerotic Cardiovascular Risk in Adults: A Report of the American College of Cardiology/American Heart Association Task Force on Practice Guidelines. J Am Coll Cardiol. 2014;63:2889–934. 10.1016/j.jacc.2013.11.002 24239923

[13] Brown WV, Clark L, Falko JM, Guyton JR, Rees TJ, Schonfeld G, et al. Optimal management of lipids in diabetes and metabolic syndrome. J Clin Lipidol. 2008;2:335–42. 10.1016/j.jacl.2008.08.444 21291758

[14] Robinson JG, Ballantyne CM, Grundy SM, Hsueh WA, Parving HH, Rosen JB, et al. Lipid-altering efficacy and safety of ezetimibe/simvastatin versus atorvastatin in patients with hypercholesterolemia and the metabolic syndrome (from the VYMET study). Am J Cardiol. 2009;103:1694–702. 10.1016/j.amjcard.2009.05.003 19539078

[15] Smith Jr SC, Allen J, Blair SN, Bonow RO, Brass LM, Fonarow GC, et al. AHA/ACC guidelines for secondary prevention for patients with coronary and other atherosclerotic vascular disease: 2006 update: endorsed by the National Heart, Lung, and Blood Institute. Circulation. 2006;113:2363–72. 10.1161/CIRCULATIONAHA.106.174516 16702489

[16] Matthews DR, Hosker JP, Rudenski AS, Naylor BA, Treacher DF, Turner RC (1985). Homeostasis model assessment: insulin resistance and beta-cell function from fasting plasma glucose and insulin concentrations in man. Diabetologia.

[17] Ballantyne CM, Abate N, Yuan Z, King TR, Palmisano J (2005). Dose-comparison study of the combination of ezetimibe and simvastatin (Vytorin) versus atorvastatin in patients with hypercholesterolemia: the Vytorin Versus Atorvastatin (VYVA) study. Am Heart J.

[18] Goldberg RB, Guyton JR, Mazzone T, Weinstock RS, Polis A, Edwards P, et al. Ezetimibe/simvastatin vs atorvastatin in patients with type 2 diabetes mellitus and hypercholesterolemia: the VYTAL study. Mayo Clin Proc. 2006;81:1579–88. 10.4065/81.12.1579 17165637

[19] Foody JM, Brown WV, Zieve F, Adewale AJ, Flaim D, Lowe RS, et al. Safety and efficacy of ezetimibe/simvastatin combination versus atorvastatin alone in adults >/=65 years of age with hypercholesterolemia and with or at moderately high/high risk for coronary heart disease (the VYTELD study). Am J Cardiol. 2010;106:1255–63. 10.1016/j.amjcard.2010.06.051 21029821

[20] Robinson JG, Ballantyne CM, Hsueh WA, Rosen JB, Lin J, Shah AK, et al. Age, abdominal obesity, and baseline high-sensitivity C-reactive protein are associated with low-density lipoprotein cholesterol, non-high-density lipoprotein cholesterol, and apolipoprotein B responses to ezetimibe/simvastatin and atorvastatin in patients with metabolic syndrome. J Clin Lipidol. 2013;7:292–303. 10.1016/j.jacl.2013.03.007 23890516

[21] Chan DC, Watts GF (2011). Dyslipidaemia in the metabolic syndrome and type 2 diabetes: pathogenesis, priorities, pharmacotherapies. Expert Opin Pharmacother.

[22] Karalis DG, Ishisaka DY, Luo D, Ntanios F, Wun CC (2007). Effects of increasing doses of atorvastatin on the atherogenic lipid subclasses commonly associated with hypertriglyceridemia. Am J Cardiol.

[23] Geiss HC, Otto C, Schwandt P, Parhofer KG (2001). Effect of atorvastatin on low-density lipoprotein subtypes in patients with different forms of hyperlipoproteinemia and control subjects. Metabolism.

[24] Kalogirou M, Tsimihodimos V, Gazi I, Filippatos T, Saougos V, Tselepis AD, et al. Effect of ezetimibe monotherapy on the concentration of lipoprotein subfractions in patients with primary dyslipidaemia. Curr Med Res Opin. 2007;23:1169–76. 10.1185/030079907x188062 17519084

[25] Winkler K, Jacob S, Muller-Schewe T, Hoffmann MM, Konrad T (2012). Ezetimibe alone and in combination lowers the concentration of small, dense low-density lipoproteins in type 2 diabetes mellitus. Atherosclerosis.

[26] Tomassini JE, Mazzone T, Goldberg RB, Guyton JR, Weinstock RS, Polis A, et al. Effect of ezetimibe/simvastatin compared with atorvastatin on lipoprotein subclasses in patients with type 2 diabetes and hypercholesterolaemia. Diabetes Obes Metab. 2009;11:855–64. 10.1111/j.1463-1326.2009.01061.x 19508464

[27] Abate N, Catapano AL, Ballantyne CM, Davidson MH, Polis A, Smugar SS, et al. Effect of ezetimibe/simvastatin versus atorvastatin or rosuvastatin on modifying lipid profiles in patients with diabetes, metabolic syndrome, or neither: results of two subgroup analyses. J Clin Lipidology. 2008;2:91–105. 10.1016/j.jacl.2008.02.002 21291725

[28] Bardini G, Giorda CB, Pontiroli AE, Le Grazie C, Rotella CM (2010). Ezetimibe + simvastatin versus doubling the dose of simvastatin in high cardiovascular risk diabetics: a multicenter, randomized trial (the LEAD study). Cardiovasc Diabetol.

[29] Conard S, Bays H, Leiter LA, Bird S, Lin J, Hanson ME, et al. Ezetimibe added to atorvastatin compared with doubling the atorvastatin dose in patients at high risk for coronary heart disease with diabetes mellitus, metabolic syndrome or neither. Diabetes Obes Metab. 2010;12:210–8. 10.1111/j.1463-1326.2009.01152.x 20151997

[30] Denke M, Pearson T, McBride P, Gazzara RA, Brady WE, Tershakovec AM (2006). Ezetimibe added to ongoing statin therapy improves LDL-C goal attainment and lipid profile in patients with diabetes or metabolic syndrome. Diab Vasc Dis Res.

